# Supplementary material for: SyNDI: synchronous network data integration framework
Source: BMC Bioinformatics. 2018 Nov 6;19:403. doi: 10.1186/s12859-018-2426-5 (PMC6219086; doi:10.1186/s12859-018-2426-5)
Supplement: Supplementary file 5 — Files of the example presented in Additional file 4. This additional file is a zip package that contains all files used in Additional file 4. Detailed descriptions of each of these files are presented in a README file included in this zip package. (GZ 30411 kb) [file 12859_2018_2426_MOESM5_ESM.gz › SyNDITools/Meme2Fimo/meme_4.9.0_4.tar.gz!/meme_4.9.0/website/html/help_width.html]

MEME Suite - Motif Width


|  |  |
| --- | --- |
|  | MEME Suite The MEME Suite web application requires the use of JavaScript   Javascript doesn't seem to be available on your browser.  - **Motif Width (MEME)**    This is the width (number of characters in the sequence pattern) of   a single motif. MEME chooses the optimal width of each motif   individually using a statistical heuristic function. You can choose   different limits for the **minimum** and **maximum** motif   widths that MEME will consider. The width of each motif that MEME   reports will lie within the limits you choose.    **Note on maximum width:**    Both protein and DNA motifs are often shorter than the   default "maximum width" (50). It is often advisable for   you to reduce that parameter to a much smaller value   (e.g., in the range 10--20) by entering a new value in the box. - **Number of aligned columns (GLAM2)**    GLAM2 allows you to set limits on the number of "key positions"--   the aligned columns--in motifs. (Because GLAM2 allows gaps,   instances of the motif may not have the same width as the number   of aligned columns.) GLAM2 automatically adjusts the number   of key positions so as to maximize the alignment score,   but it sometimes has trouble with this. You can help GLAM2 by   changing the initial number of aligned columns to a ballpark   value. You can also set lower and upper bounds on the   number of aligned columns in the motif.    **Note on maximum number of aligned columns:**    Both protein and DNA motifs are often shorter than the   default "maximum number of aligned columns" (50).   It is often advisable for   you to reduce that parameter to a much smaller value   (e.g., in the range 10--20) by entering a new value in the box. |
